# Sky island bird populations isolated by ancient genetic barriers are characterized by different song traits than those isolated by recent deforestation

**DOI:** 10.1002/ece3.2475

**Published:** 2016-09-22

**Authors:** Chetana B. Purushotham, V. V. Robin

**Affiliations:** ^1^ National Institute of Advanced Studies Indian Institute of Science Campus Bangalore India; ^2^ Present address: V. V. Robin, Indian Institute of Science Education and Research Tirupati Tirupati India

**Keywords:** bird song, endemic, shola, sky islands, songbird, Western Ghats

## Abstract

Various mechanisms of isolation can structure populations and result in cultural and genetic differentiation. Similar to genetic markers, for songbirds, culturally transmitted sexual signals such as breeding song can be used as a measure of differentiation as songs can also be impacted by geographic isolation resulting in population‐level differences in song structure. Several studies have found differences in song structure either across ancient geographic barriers or across contemporary habitat barriers owing to deforestation. However, very few studies have examined the effect of both ancient barriers and recent deforestation in the same system. In this study, we examined the geographic variation in song structure across six populations of the White‐bellied Shortwing, a threatened and endemic songbird species complex found on isolated mountaintops or “sky islands” of the Western Ghats. While some sky islands in the system are isolated by ancient valleys, others are separated by deforestation. We examined 14 frequency and temporal spectral traits and two syntax traits from 835 songs of 38 individuals across the six populations. We identified three major song clusters based on a discriminant model of spectral traits, degree of similarity of syntax features, as well as responses of birds to opportunistic playback. However, some traits like complex vocal mechanisms (CVM), relating to the use of syrinxes, clearly differentiated both ancient and recently fragmented populations. We suggest that CVMs may have a cultural basis and can be used to identify culturally isolated populations that cannot be differentiated using genetic markers or commonly used frequency‐based song traits. Our results demonstrate the use of bird songs to reconstruct phylogenetic groups and impacts of habitat fragmentation even in complex scenarios of historic and contemporary isolation.

## 1 Introduction

Breeding song is a social trait essential for individual recognition amongst songbirds, especially between conspecifics when establishing territories and choosing mates (Gil & Gahr, 2002; Koetz, Westcott, & Congdon, 2007; Lynch, 1996). As with genes, the transmission of songs between individuals of a population can be affected by geographic separation (Baker, Baker, & Tilghman, 2006; Koetz et al., 2007; Lynch, 1996). In isolated populations, novel song elements can accumulate and, over time, result in geographic variation in song structure within a species (Catchpole & Slater, 1995). Significant differences in song structure can form behavioral barriers between individuals leading to premating reproductive isolation (Irwin, Bensch, & Price, 2001; Podos & Warren, 2007), making song divergence one of the essential components of speciation in songbirds (Lachlan & Servedio, 2004; Price, 2008). Identifying song differences and the barriers to song transmission can, therefore, help identify barriers to dispersal and connectivity between populations (Kirschel, Blumstein, & Smith, 2009; Slabbekoorn & Smith, 2002).

Studies of song variation (based on spectral characteristics or degree of note, syllable, or song sharing) have indicated that isolation over evolutionary time scales can lead to song divergence (Baker et al., 2006; Päckert & Martens, 2004; Price & Lanyon, 2002). Other studies also suggest that contemporary changes in habitat such as deforestation can also act as barriers, resulting in song differentiation at much shorter time scales (Laiolo & Tella, 2007; Osiejuk, Ratyńska, Cygan, & Dale, 2003; Parker, Anderson, Jenkins, & Brunton, 2012). However, few natural systems allow an examination of the impacts of both contemporary fragmentation and historical isolation on song evolution (Potvin & Clegg, 2015). Such a study could identify the spatial and temporal scales at which important song traits, such as song frequency or song complexity, begin to differentiate. Comparing the effects of ancient and recent isolation on the evolution of such song traits may also be useful in understanding songbirds in fragmented landscapes, where differentiation of specific song traits can potentially indicate how songbird populations are responding to the effects of habitat fragmentation and loss of connectivity (Laiolo & Tella, 2006).

Studies of song variation and effects of habitat modification have largely been from the temperate regions (Podos & Warren, 2007). In temperate regions, habitat structure and species communities are relatively simple (both factors known to influence song evolution) (Weir & Wheatcroft, 2011) compared to the tropics (Singh & Price, 2015). Tropics have also experienced higher rates of contemporary habitat deforestation than the temperates (Dirzo & Raven, 2003; Ribeiro, Metzger, Martensen, Ponzoni, & Hirota, 2009; Sodhi et al., 2010). In temperate songbird species such as the Dupont's Lark (*Chersophilus duponti*), habitat fragmentation is known to have led to a decrease in syllable diversity and song repertoires, leading to cultural bottlenecks and reduced viability in populations (Laiolo & Tella, 2006). Such studies, however, have rarely been conducted in the tropics, particularly in the Old World. Further, cultural differentiation can be exemplified by the plasticity and variability of song structure seen in oscine species (many are open‐ended song learners) (Podos & Warren, 2007), many of which inhabit the tropics (Fjeldså, 2013).

Here, we studied song divergence in an oscine songbird species complex, the White‐bellied Shortwing (WBS) or the White‐bellied Blue Robin (Rasmussen & Anderton, 2012) (taxonomically two species *Myiomela albiventris* and *M. major*) in a montane landscape, to understand the effects of multiple scales of isolation. We compared historical isolation (millions of years) across geographically isolated mountaintops or “sky islands” (Robin, Vishnudas, Gupta, & Ramakrishnan, 2015b), and recent isolation (100–150 years) within sky islands due to deforestation (Robin, Gupta, Thatte, & Ramakrishnan, 2015a). Using spectral‐ and syntax‐based song traits, we asked (a) what spatial and temporal scales of isolation lead to song differentiation in this landscape, (b) what song traits correlate with isolation at these different scales, and (c) what aspects of isolation (distance or barriers) influence song differentiation and the correlation of song and genetic differentiation. Spectral traits are known to evolve more rapidly than syntactical traits (Podos & Warren, 2007), and due to the plasticity in the WBS song (Robin, Katti, Purushotham, Sancheti, & Sinha, 2011), there is potential for variations to accumulate rapidly in isolation. We expected to find song differentiation between different populations, perhaps more due to differences in spectral song traits. While some studies have shown patterns of song and genetic differentiation to be correlated (Irwin, Thimgan, & Irwin, 2008; Potvin & Clegg, 2015), in this system, we expected that a relationship between song and genetic differentiation (*F*_ST_), if any, could be a result of the effect of geographic isolation (geographic distance and ancient barriers) driving both.

## 2 Methods

### 2.1 Study area

Shola forests, or tropical montane cloud forests (Bunyan, Bardhan, & Jose, 2012), are typically found above 1,400 m ASL on the mountain tops or “sky islands” of the Western Ghats. Each sky island is isolated from neighboring islands by deep valleys characterized by different climate and vegetation, described in Robin et al. (2015b). The WBS, an IUCN listed “endangered” understory songbird (BirdLife International, 2012), is most easily located and identified in dense shola forests by its distinctive breeding song—known to be complex and highly variable (Robin et al., 2011).

### 2.2 Sampled populations

**Figure 1:**
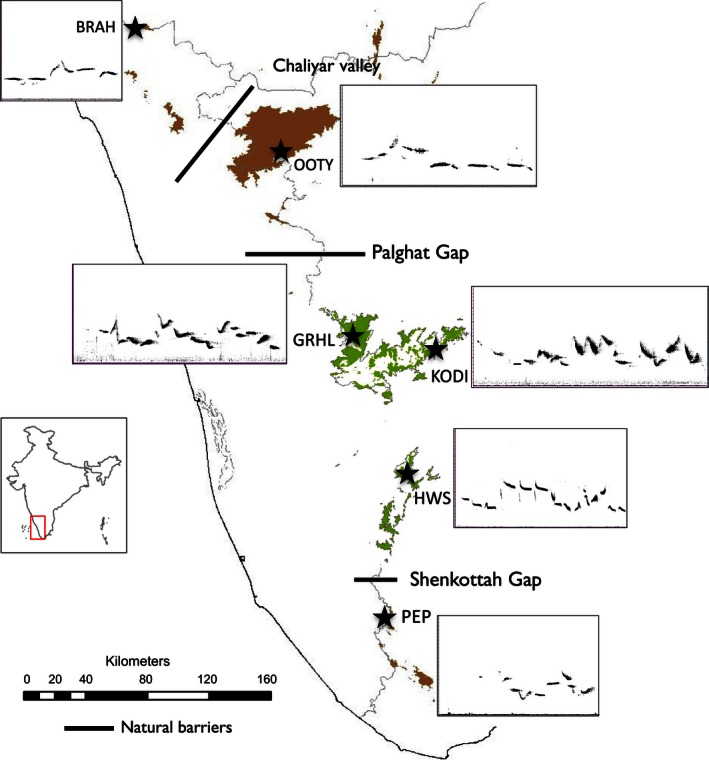
A map of the montane shola sky island system in the Western Ghats of India highlighting the six study populations of the White‐bellied Shortwing (WBS) complex. BRAH—Brahmagiri and OOTY—Ooty are currently *Myiomela major,* and GRHL—Grasshills, KODI—Kodaikanal, HWS—Highwayvis, and PEP—Peppara are *M*. *albiventris* (Rasmussen & Anderton, 2012). Insets next to each location depict the spectrogram of a song from that population (high‐resolution spectrograms in the Supporting Information Fig. S1). Solid black lines show the three known natural genetic barriers to the WBS. Green patches in the central sky islands indicate fragmented forests

Our study was conducted on six geographically isolated populations of WBS (Brahmagiri—BRAH, Ooty—OOTY, Kodaikanal—KODI, Grasshills—GRHL, Highwayvis—HWS, and Peppara—PEP), on four sky islands largely north–south oriented, along the Western Ghats of India, covering almost the entire distributional range (~600 km) of the species complex (Figure 1). Each population is located on a different sky island, except Kodaikanal, Grasshills, and Highwayvis that lie on the central Anamalais sky islands. The Palghat Gap is a known genetic barrier, resulting in the deepest divergence in the WBS (4.9 MYA) followed by another divergence across the Shencottah Gap (1.6 MYA) (Robin, Sinha, & Ramakrishnan, 2010). Populations of WBS from Brahmagiri and Ooty have also diverged on either side of the Chaliyar River for approximately several thousand years [based on Robin et al., 2010, 2015a]. In contrast, the three populations within the central Anamalais sky island have only been recently isolated from each other (100–150 years) due to recent deforestation for cultivation (85% of the landscape), resulting in lower contemporary gene flow compared to historic gene flow (Robin et al., 2015a). The six study populations, therefore, fall along a gradient of geographic isolation and genetic differentiation, with some populations historically diverged across major geographic gaps or barriers in the landscape, while others show reduced gene flow corresponding with recent isolation by deforestation.

### 2.3 Song sampling and analysis

Field recording of WBS song was conducted over 3 years (2007, 2008, and 2011) between January and April when territorial males are known to sing (Robin et al., 2011). At all locations, sampling was carried out within a narrow elevation range of 1,400–1,900 m above sea level, ensuring reasonable similarity in habitat structure and bird community composition.

Field recording protocols followed and are detailed in Robin et al. (2011) and the Supporting Information. Analog recordings were digitized in Raven Pro 1.4 (Bioacoustics Research Program, 2011) and converted into spectrograms (Hann window‐500 samples) (Bioacoustics Research Program, 2011). For each song, we collected data on (a) spectral and (b) visually assessed song complexity variables. For the first set of variables, we used the “start time”, “end time”, “low frequency”, and “high frequency” variables from RAVEN for each “note”—a continuous trace on the spectrogram (Gil & Gahr, 2002). Using these basic variables, we derived measurements at the song level (where song is a group of notes separated distinctly in time (Koetz et al., 2007)).

### 2.4 Spectral traits

We measured the fundamental frequency and time duration of each note and related measures using Raven Pro 1.4 and subsequently computed mean and variance measurements at the song level. For each song, we also quantified spectral variables indicative of one of two potential complex vocal mechanisms (CVM): (1) the “two‐voice phenomenon”—simultaneous usage of both sides of the syrinx (the paired vocal apparatus in birds) referred to in this article as “dual syrinx usage” or (2) the “nonlinear phenomena” involving nonlinear dynamics of a single syrinx (Zollinger, Riede, & Suthers, 2008). To do this, we measured two variables, the number of occurrences of (i) internote intervals <2 ms (indicative of notes being sung below the minimum duration known for a “minibreath” to take place between notes (Suthers, 2004)) and (ii) temporal overlap between two consecutive notes as these CVMs can allow birds to simultaneously produce more than one note at different frequencies (Suthers, 2004). Both variables were scaled by the total number of notes in a song to remove any bias resulting from population‐level differences in the number of notes per song. While we cannot distinguish between which of the two CVMs are at play, patterns observed here offer a preliminary understanding to further investigate these underlying mechanisms. Biologically these CVMs are interesting as they allow songbirds to increase the complexity and variety of their songs (Suthers, 2004; Zollinger et al., 2008). In total, we derived 14 spectral variables which were used for further analysis. All spectral variables and their definitions are listed in Table S1 of the Supporting Information.

### 2.5 Syntax complexity

We measured two visually estimated variables from a subset of 11 consecutive songs from each individual in the dataset. Eleven was the minimum number of consecutive songs available for some individuals, and in order to use similar sample sizes across individuals, we used consecutive sets of 11 songs per individual. We used a bout with consecutive songs to assess versatility within bout based on Singh & Price (2015). With these data, we first computed a Song Variety Index (SVI) [based on Singh and Price (2015)], as the ratio of unique songs or “song types” to the total number of songs (*N* = 11) sung by an individual in a sequence or song bout. Second, as a measure of versatility in song organization (the arrangement of songs within a song bout), for each bout (*N* = 11) of an individual, we estimated the proportion of songs that were (a) completely new—”new”, if altered by the absence or addition of at least one note, (b) modified from previous songs—”mod”, if the song was new but containing a combination of notes exactly copied from a previous song, (c) already sung at least once but not in succession—”old”, and (d) old songs repeated in succession—”same”. However, differences in frequency and change in number of notes due to a shortened or elongated trill did not constitute a new song (Singh & Price, 2015). To ascertain whether estimates of syntax complexity are affected by the number of consecutive songs examined (11 in this study), we repeated the SVI analysis on two individuals from the GRHL and OOTY populations, increasing the sample size to 27. To check for bias in the estimate of SVI due to the use of songs that are in sequence, we calculated SVI on multiple song sequences using random start points. Visual identification and these analysis were performed by CBP; however, to check for bias, a subset of the entire dataset was analyzed blind by both authors independently and found to be consistent in their identification. We generated a song type library with unique song types across individuals of all populations to examine the degree of song sharing between individuals within and across study populations.

### 2.6 Data analysis

We examined a total of 835 songs from 38 individuals across the six populations, with a mean of six individuals sampled per population (range 2–10). The BRAH sky island which is represented by two individuals in the dataset is from a small, isolated, northern distribution limit, and a small population of WBS. For each individual, a mean ± *SD* of 22 ± 7 songs was recorded (range 11–30). Visual assessment of song bouts to quantify syntax complexity generated a song type library of 270 unique song types from the 418 songs that were analyzed (11 songs from each of 38 individuals). In all populations, we observed no instance of sharing of a song type, neither across individuals of different populations nor between individuals of the same population. In other words, each individual had a unique set of songs. Repetition of song types was only observed within an individual song bout.

We reduced the 14 spectral variables to five uncorrelated principal components (PRIN) (eigenvalues >1, 78.2% of variation) for further analyses (detailed in Supporting Information). We tested for differences in song within and across populations using a nested (songs, individuals, and populations) multivariate analysis of variance (MANOVA). We also conducted a nested analysis of variance (ANOVA) on each PRIN variable separately to examine which variables led to song differences between and within populations. Further, we conducted a discriminant function analysis (DFA) using the five principal components to classify songs to their populations based on spectral variables. We repeated the analyses of song differences between populations using a nonmetric multidimensional scaling analysis (NMDS) based on all the 14 variables as an NMDS is at times thought to be better suited for data of different scales (Koetz et al., 2007).

We tested for population differences in the two measures indicative of dual syrinx usage, using a Kruskal–Wallis rank‐sum test and Dunn's Kruskal–Wallis multiple comparisons test (with a Bonferroni correction). To examine whether increase in syrinx usage is correlated with greater shifts in frequency between notes (which is expected to increase complexity of the song), we used a two‐sample Kolmogorov–Smirnov test to compare frequency shifts (computed as the difference between the high frequency of a note and the low frequency of the preceding note for each note) between pairs of consecutive notes with internote intervals > and < than 2 milliseconds (ms) in each song. Additionally, to compare populations based on the measures of syntax complexity, we computed the mean and bootstrapped confidence intervals (1,000 replications) for the two variables—SVI and versatility in song organization. We also examined (using a Pearson correlation) if degree of complexity of a song (SVI) is influenced by its spectral characteristics (song frequency bandwidth and song delivery rate—two important frequency and time‐derived characteristics of song based on our previous analyses).

### 2.7 Measuring isolation

We examined the effect of geographic isolation on the patterns of song differentiation, testing for spectral and syntax traits separately. We performed Mantel and partial Mantel tests to examine whether song differences between populations were influenced differently by the different mechanisms of geographic isolation (geographic distance and the presence of barriers) and to examine how these correlated with genetic differentiation. Methods used to compute pairwise distances between populations are detailed in the Supporting Information.

The PCA, DFA, and MANOVA were performed in JMP version 8.0 (1989), and all remaining statistical analyses and data visualizations were performed in R version 3.1.2 (R Core Team, 2014).

## 3 Results

### 3.1 Where does song differentiation occur?

**Figure 2:**
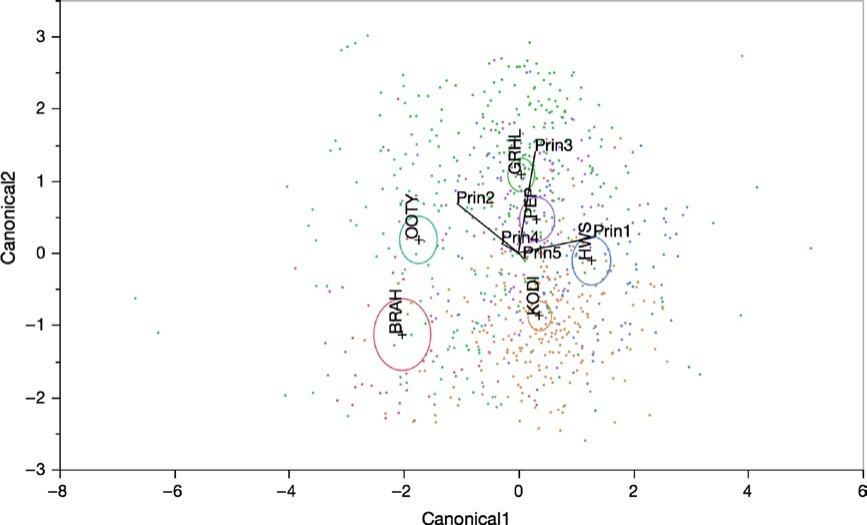
Canonical plot of songs from the six populations with spectral data from the five reduced variables (principal components Prin1 to Prin5) represented with points and color‐coded 95% confidence ellipses. Canonical correlation for the two composite functions = .68, .62, respectively. BRAH: Brahmagiri, OOTY: Ooty, GRHL: Grasshills, KODI: Kodaikanal, HWS: Highwayvis, PEP: Peppara

We found that songs sampled from all six populations were significantly different in their spectral variables, with the largest differences between populations (MANOVA: *F* = 58.546, *p* < .0001) and to a lesser extent within a population (MANOVA: *F* = 5.779, *p* < .0001). These differences were further substantiated when examined in multivariate space using a discriminant model (Figure 2). Two canonical discriminant functions (canonical correlation = .68, .62, respectively) contributed significantly toward separating songs from the six populations (correctly classifying 57.8%).

Although all six populations had different song structure, the most difference (along Canonical1) was between Ooty–Brahmagiri and the Anamalais, which lie on either side of the Palghat Gap and are genetically most distinct. Differences along the first canonical function (variance explained = 53%, canonical correlation = .68) were correlated with PRIN1 (mean and *SD* of high frequency, mean and *SD* of note bandwidth, song bandwidth, and *SD* of low frequency) and PRIN2 (mean and *SD* of note length and delivery rate). Ooty and Brahmagiri were characterized by spectrally simple songs, composed of slow, long notes and narrow note and song frequency bandwidth. In contrast, songs of Anamalais (Grasshills, Kodaikanal, and Highwayvis) were complex, characterized by short notes, with high note and song frequency bandwidth, sung at fast song delivery rates (Table S3 in Supporting Information).

The first canonical function (correlated with PRIN1 and PRIN2) also differentiated song between the Anamalais and Peppara, although to a lesser degree. These populations are geographically and genetically isolated by the second of the two ancient barriers, the Shencottah Gap. The songs from Peppara differed from the Anamalais and other populations in having songs of an intermediate spectral complexity, that is, intermediate note and frequency bandwidth, sung at intermediate delivery rates.

**Figure 3:**
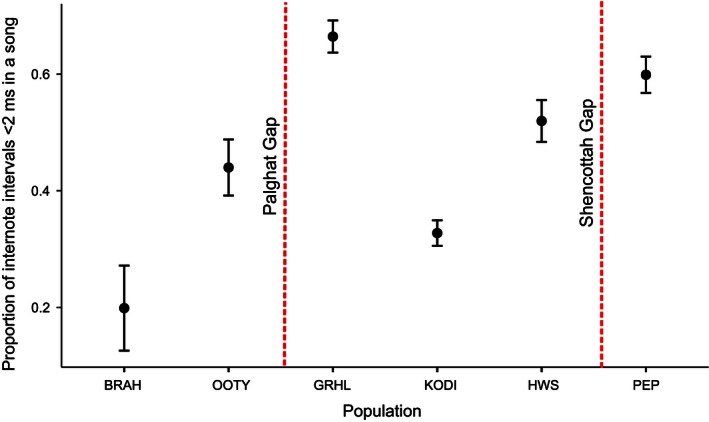
All six WBS populations (represented from north to south on x‐axis) significantly differ in the usage of the CVMs (mean occurrences of consecutive notes with internote intervals <2 ms) per song (Kruskal–Wallis Χ^2^ = 315.95, *df* = 5, *p* < .0001, Dunn's test results in Supporting Information results). Error bars represent 95% confidence intervals around the mean. BRAH: Brahmagiri, OOTY: Ooty, GRHL: Grasshills, KODI: Kodaikanal, HWS: Highwayvis, PEP: Peppara

The second canonical function (variance explained = 39.6%, canonical correlation = .62) was strongly correlated with PRIN3 (the two syrinx variables). This was able to further discriminate between populations across relatively recent barriers separating genetically divergent Ooty and Brahmagiri populations across the Chaliyar River as also the genetically similar but recently fragmented populations within the Anamalais–Grasshills, Kodaikanal, and Highwayvis. Populations within each of these clusters were found to differ significantly in the internote intervals <2 ms per song (Figure 3), and number of temporal note overlaps (Fig. S4 in Supporting Information). This suggested significant differences between populations in the degree of usage of the CVMs in their songs (spectrogram in Supporting Information, Fig. S2). We also found evidence that the range of frequency shifts are greater when internote intervals are <2 ms (less than a minibreath) than when internote intervals are >2 ms (two‐sample KS test: D = 0.16, *p* < .001).

These relative differences in song structure between populations were consistent with a nonmetric multidimensional scaling analysis (NMDS, stress = 0.2056) on the same dataset using all 14 spectral variables (Fig. S4 in Supporting Information).

### 3.2 Differences in song syntax complexity

**Figure 4:**
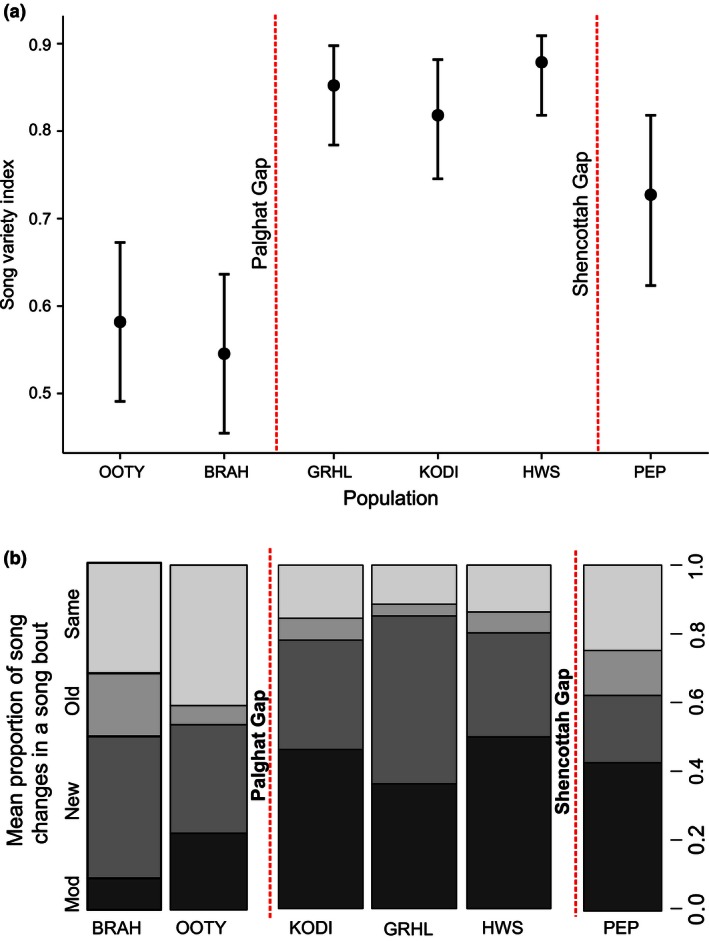
(a) Variation in the Song Variety Index (SVI) between populations (represented from north to south on x‐axis) forming three clusters, corresponding to genetic clusters isolated by the ancient Palghat Gap and Shencottah Gap. Error bars indicate 95% confidence intervals. BRAH: Brahmagiri, OOTY: Ooty, GRHL: Grasshills, KODI: Kodaikanal, HWS: Highwayvis, PEP: Peppara. (b) Mean proportion of song changes in a bout indicating differences in versatility in song organization between populations. Songs were categorized as new = completely new songs, “mod” = modified from previous songs in the same bout, “old” = already sung in the bout, and/or “same” = remained unchanged from the song preceding it. BRAH: Brahmagiri, OOTY: Ooty, GRHL: Grasshills, KODI: Kodaikanal, HWS: Highwayvis, PEP: Peppara. Plot with error bars available in Fig. S5 of Supporting Information

Syntax complexity based on the Song Variety Index (SVI) and the versatility in song organization did not differ between all pairs of populations and only between populations separated by the two most ancient barriers—Palghat and Shencottah gaps (Figure 4a,b). The Anamalais population had the highest SVI (GRHL: 0.85 ± 0.10 *SD*, KODI: 0.81 ± 0.11 *SD*, HWS: 0.87 ± 0.07 *SD*) followed by PEP (0.72 ± 0.14 *SD*) and the northern populations (OOTY: 0.58 ± 0.12 *SD* and BRAH: 0.54 ± 0.12). We also found SVI to be strongly correlated with song frequency bandwidth (Pearson's correlation r = .53, *df* = 36, *p* < .05) and delivery rate (Pearson's correlation, r = .50, *df* = 36, *p* < .05), indicating that individuals with a higher frequency bandwidth and faster song delivery rates had a larger song variety than individuals with a lower frequency bandwidth and slower song delivery rates.

The individuals from the complex song cluster (Anamalais), which possessed spectrally complex song characteristics (Table S3) on average, sang more modified songs and were less repetitive in comparison with the simple song cluster (Ooty and Brahmagiri), which on average sang more repeats than modified songs per bout (Figure 4b). The intermediate song cluster (Peppara) was characterized by moderate spectral complexity. Individuals from Peppara, on average, had more song repetitions per song bout than the complex Anamalais cluster but still had more modified songs per bout than the simple cluster (Ooty and Brahmagiri).

In the analysis performed to check for bias, we found that the SVI calculated with only one individual each of a population with 27 consecutive recordings (GRHL, OOTY) as well as with random start points (GRHL = 0.92, OOTY = 0.54) still fell within population estimates of the SVI (GRHL–OOTY). The patterns of SVI differences across these populations, thus, could be recovered even with a single individual.

### 3.3 What aspect of isolation impacts song differentiation?

We found a strong correlation between song differences (measured using spectral and syntax variables, separately) and geographic isolation between populations (geographic distance and the presence of ancient barriers, separately). While spectral differences correlated only with geographic distance (Mantel test: r = .76, *p* = .001) and not with the presence of barriers (r = .66, *p* = .06), differences in syntax complexity correlated with both geographic distance (r = .57, *p* = .04) and with barriers (r = .88, *p* = .004). This suggests that different song traits respond differently to the same parameters of isolation.

Song differences were highly correlated with genetic differences (spectral traits: r = .84, *p* = .03; and syntax traits: r = .90, *p* = .002). When geographic distance was controlled, genetic and spectral differences were no longer correlated (partial Mantel test: r = .35, *p* = .18). When barriers were controlled, they remained correlated (partial Mantel test: r = .85, *p* = .003). Contrastingly, the correlation between genetic and syntax differences was not significant after controlling for barriers (Syntax: r = .50, *p* = .06), while remaining unaffected when controlled for geographic distance (Syntax: r = .88, *p* = .006). These analyses suggest that spectral traits were good indicators of differences due to geographic distance, and syntax differences were primarily across ancient barriers.

## 4 Discussion

We examined the contrasting influences that multiple scales of isolation have had on song differentiation using a suite of song traits representing spectral and syntax features of bird song. We found that all six study populations were different from each other when spectral traits were examined in a multivariate space. These findings corroborate other studies that have found that spectral song traits can lead to differentiation between songs due to their rapidly evolving nature (Osiejuk et al., 2003), thereby allowing us to potentially investigate genetic boundaries between populations (Podos, 2007).

We found the spectral differentiation between populations to also be correlated with genetic differentiation. While this does not suggest that song differentiation is driven by genetic differentiation, the correlation of spectral song differences with geographic distance suggests that both song and genetic differentiation are influenced by the effects of isolation and lack of connectivity across populations. There could also be other drivers such as morphological variation that can drive both song and genetic divergence between populations (Potvin & Clegg, 2015). These remain unexamined in the current study although there is some unpublished, preliminary evidence of the populations of the three song groups differing in morphology.

Our results also show that CVMs, which are known to increase the complexity of song, are able to distinguish populations across historic and recent isolation. CVMs, either the “two‐voice phenomenon” or the “nonlinear phenomena (NLP)”, are both known to allow birds to increase the complexity of songs by facilitating temporal overlaps in notes and increasing the range of frequencies they can shift between notes (Zollinger et al., 2008). We found evidence for this in the WBS as well (spectrogram in Fig. S2). Although such complex use of the syrinx has been documented in many species (including the northern cardinal*—Cardinalis cardinalis*, brown‐headed cowbird—*Molothrus ater* (Suthers, Goller, & Pytte, 1999), wood thrush—*Hylocichla mustelina* (Kroodsma, 2005), and northern mockingbird—*Mimus polyglottos* (Zollinger et al., 2008)), this may be the first documentation of such differences between populations. It may be argued that the different levels of prevalence of CVMs in some populations—high in Highwayvis and Grasshills while low in Brahmagiri and Kodaikanal—may be genetically determined as with other traits that affect song, such as body mass (Forstmeier, Burger, Temnow, & Derégnaucourt, 2009). Alternatively, these may also be culturally transmitted between individuals within a population, indirectly from song tutor to learner if imitating a tutor's repertoire of songs is impossible without the use of CVMs.

Despite differences in all six populations in specific traits (such as syrinx usage) and minor variation in overall spectral structure, song differentiation in the WBS is most clearly observable across the ancient geographic barriers (Palghat Gap, followed by the Shencottah Gap). Neither the Chaliyar River nor the recent fragments have led to very significant overall song variation.

Synthesizing all information from the discriminant model and other analyses, we grouped the six populations under three “song clusters”, with individuals from populations within each cluster sharing salient features of spectral song structure. These are the (1) simple song structure of Ooty–Brahmagiri, (2) complex structure of Anamalais, and (3) the intermediate structure of Peppara. This is further corroborated by the differences in syntax‐based song structure measured by the SVI and versatility in song organization. Syntax traits such as versatility in song organization have been thought to be evolutionarily less labile and more likely to be conserved over time compared to spectral traits (Osiejuk et al., 2003; Podos & Warren, 2007). In the WBS, differences in syntax correlated with the presence of geographic barriers.

Our identification of three biological song clusters is also supported by the response of birds to play back. Although we had no systematic experiment examining responses of individuals (Päckert & Martens, 2004) to the playback of songs from each of the study populations, we had conducted opportunistic playback trials during mist netting (Robin et al., 2010, 2015a, 2015b) that provide qualitative evidence for the existence of three predominant song clusters. Populations promptly responded (rapid capture in the mist net) to songs played back from their own population [as in 40]. Prompt responses were also observed in the populations within the Anamalais, separated only by fragmentation (Grasshills, Kodaikanal, and Highwayvis) when played back songs recorded from each other. The same was observed between populations separated by the Chaliyar River (Ooty–Brahmagiri). Contrastingly, songs from Anamalais received no response from either side of the Palghat Gap (and vice versa), and individuals of Peppara, south of the Shencottah Gap, had a much delayed response to Anamalais songs (these need to be verified with control experiments).

There are a number of competing hypotheses on which factors may be driving differences in overall spectral and syntax complexity. For instance, a recent study suggests that sequential colonization of an island chain resulted in a progressive loss of song structure of Chaffinches in the Atlantic islands (Lachlan et al., 2013). Other studies have suggested the importance of climate variability, acoustic properties of the environment, male competition, or seasonality in food [reviewed in 20], which need to be tested in this system. However, the spatial distribution of the clusters across the range of WBS, in addition to the similarity in the shola habitat structure and bird communities, leads us to speculate that geographic variation in this species may be due to drift. We expect that interplay between three factors, (a) cultural or genetic drift from a pool of highly plastic song traits, (b) a species complex with limited dispersal, and (c) a history of isolation, may have resulted in the three different trajectories of song complexity. However, these differences in complexity may still have a bearing on the further evolution of populations. For instance, song structure from populations such as Ooty and Brahmagiri continues to be similar despite being separated by a river barrier for thousands of years. We suggest that this could be explained by the spectral simplicity and low versatility of the song allowing for relatively lesser lability to change with time, even after isolation across barriers. On the contrary, the populations in the complex song cluster separated by recent deforestation and reduced gene flow for only 100–150 years (Anamalais) already exhibit minor differences in overall spectral structure. We anticipate that persistent fragmentation over time can create further isolation between these populations in the Anamalais and the relatively higher complexity and versatility of their songs may exaggerate the present variation in spectral and temporal traits toward divergence. Consequently, these fragmented populations could become smaller, accelerating both song and genetic differentiation and reducing future population viability, if habitat connectivity is not restored—a feasible option in this landscape.

In conclusion, our results reveal major song divergence across ancient geographic and genetic barriers, followed by lower levels of song differentiation between fragmented populations known to experience lower contemporary gene flow over the past century. These comprehensive patterns of song differentiation are also able to provide information on the population history of WBS, at a resolution similar to genetic markers. We propose that different song traits (including rapidly evolving or conserved) can act as reliable preliminary markers to reconstruct phylogenetic population groups in oscine songbirds. Further, we suggest that the transmission of CVMs may have a cultural basis, and prove to be higher resolution markers than genetic and regular frequency‐based song traits in detecting culturally isolated populations. Complementing genetic markers and ecological parameters, songs could also be used to predict future population trajectories in the tropics and population viability in fragmented landscapes, as has been carried out in temperate regions (Laiolo & Tella, 2006). In doing so, we may contribute to the conservation of songbird species in a rapidly changing world.

## Conflict of Interest

None declared.
