# Supplementary material for: Sky island bird populations isolated by ancient genetic barriers are characterized by different song traits than those isolated by recent deforestation
Source: Ecol Evol. 2016 Sep 22;6(20):7334–43. doi: 10.1002/ece3.2475 (PMC5513277; doi:10.1002/ece3.2475)

**Supplementary Material**

Fig. S1: Spectrograms of White-bellied Shortwing (WBS) species complex songs from each of the six sampled populations

***Supplementary Methods***

Table S1: Spectral variables obtained for each of the WBS songs using Raven Pro 1.4

| **Variable** | **Description** |
| --- | --- |
| Mean low frequency | Mean of low frequency measurements of all notes in a phrase |
| Mean High Frequency | Mean of high frequency measurements of all notes in a phrase |
| Mean Note Bandwidth | Mean of delta frequency (high frequency-low frequency) of all notes in a phrase |
| Mean Note Length | Mean of the lengths of all notes in a song |
| SD Low Frequency | Standard deviation from mean of low frequency measurements of all notes in a song |
| SD High Frequency | Standard deviation from mean of high frequency measurements of all notes in a song |
| SD Note Bandwidth | Standard deviation from mean of delta frequency (high frequency-low frequency) of all notes in a song |
| SD Note Length | Standard deviation from mean of the lengths of all notes in a song |
| Notes Per Song | Number of notes in a song |
| Song Length | The length of a song from the start of the ﬁrst note to the end of the last note |
| Song Bandwidth | Difference between the highest and lowest frequencies of all notes in a song |
| Delivery rate | Number of notes in a song per second |
| No. of inter-note intervals < 2 ms per song | Number of inter-note intervals that were less than 2 ms in duration per number of notes in a song |
| No. of temporal note overlaps per song | Number of temporal note overlaps per number of notes in a song |

*Song Sampling*

Field recording of individual singing males was carried out with a Sennheisser shotgun microphone (ME66 – K6) on Marantz PMD222 or Marantz PMD660 recorder between 0700 to 1100 hours each morning. Each recording consisted of continuous song bouts from a single individual recorded at a distance of 8- 20 meters. In one population (Grasshills), individuals were colour banded and identity could be confirmed visually, while in other locations distance between individuals (~50-100m, based on VVR’s field experience and territory size information from a mark-recapture study), and simultaneous singing of neighbouring individuals, were used as a proxy to avoid recording the same individual twice.

Analog recordings were digitized and converted into spectrograms in Raven Pro 1.4 [1] at a sampling rate of 48 kHz. Songs with poor recording quality and high background noise from rain, vehicles and other species vocalisations were removed from the analyses. For each song, we collected data on a) spectral variables, quantified based on frequency and time measurements and b) visually assessed song complexity variables. For the first set of variables, we used the ‘start time’, ‘end time’, ‘low frequency’ and ‘high’ frequency variables from RAVEN for each ‘note’ - a continuous trace on the spectrogram [2]. Using these basic frequency and time-based measures for each note, we derived measurements that we expected to be important in differentiating song structure between populations at the song-level (where song is a group of notes separated distinctly in time [3]).

*Data Analysis*

*Principal Components Analysis*

The spectral variables with the maximum loading onto each of the five chosen PRIN scores were – PRIN1: mean high frequency, standard deviation (SD) of low frequency, note bandwidth (mean and SD) and song bandwidth; PRIN2: note length (mean and SD) and song delivery rate; PRIN3: temporal note-overlap and inter-note interval <2 ms; PRIN4: mean low frequency; and PRIN5: song length.

Table S2: Principal component scores for the spectral variables for the WBS used in the final analysis

| **Prin1** | **Prin2** | **Prin3** | **Prin4** | **Prin5** | **Prin6** |
| --- | --- | --- | --- | --- | --- |
| **no. of notes** | 0.23747 | -0.27614 | 0.15004 | -0.11049 | 0.45634 |
| **mean note length** | -0.17389 | 0.57137 | 0.05106 | 0.00982 | 0.08752 |
| **StdDev of note length** | 0.05362 | 0.42873 | 0.07284 | -0.14534 | 0.0644 |
| **mean low freq** | 0.0612 | 0.09512 | -0.11278 | 0.73316 | 0.03237 |
| **StdDev of Low Freq** | 0.33042 | -0.07227 | 0.0216 | 0.30165 | 0.12384 |
| **mean High Freq** | 0.30797 | 0.19847 | -0.28409 | 0.3663 | -0.15097 |
| **StdDev of High Freq** | 0.4076 | 0.07914 | -0.094 | 0.01123 | 0.07808 |
| **mean note bandwidth** | 0.33294 | 0.15657 | -0.24892 | -0.27733 | -0.22631 |
| **StdDev of note bandwidth** | 0.32239 | 0.17919 | -0.24031 | -0.32946 | -0.13622 |
| **song length** | -0.00566 | 0.14612 | -0.05777 | -0.03322 | 0.76897 |
| **song bandwidth** | 0.45132 | -0.01219 | 0.00135 | -0.10555 | 0.16239 |
| **delivary rate** | 0.25296 | -0.42537 | 0.24786 | 0.0228 | -0.16584 |
| **mini breath per no. of notes** | 0.1764 | 0.19974 | 0.58647 | 0.01748 |  |
| **overlap per no. of notes** | 0.15158 | 0.23365 | 0.58174 | 0.07231 | -0.09937 |

*Mantel Tests*

Song distance was measured and tested separately for the spectral and syntax traits. We used the mean pair-wise Euclidean distance between pairs of populations centroids based on the five PRIN scores to compute song distance (spectral and SVI) using the vegdist function in the vegan package,[4]). Geographic distance between pairs of populations was measured as the straight line distance between their location centres on a map. Presence of geographic barriers, tested separately, was treated as an indicator matrix (in IBDWS version 3.23 [5]), scoring each on a scale of 1 to 10 based on the degree to which each acts as a barrier to gene flow [6], Palghat Gap =10, Shencottah Gap=7, Chaliyar River =2 and contemporary fragmentation=1 [6,7]. We also examined whether patterns of song differentiation were correlated with genetic differentiation (measured using pair-wise F_st_ [6,7]), while also controlling for the effect of geographic isolation.

References

1. Bioacoustics Research Program. 2011Raven Pro: Interactive sound analysis software (Version 1.4) [computer software].Ithaca, NY: The Cornell Lab of Ornithology. Available from <http://www.birds.cornell.edu/raven>
2. Singh, P. & Price, T.D. 2015 Causes of the latitudinal gradient in birdsong complexity assessed from geographical variation within two Himalayan warbler species. *Ibis* (doi:10.1111/ibi.12271)
3. Robin, V.V., Katti, M., Purushotham, C., Sancheti, A. & Sinha, A. 2011 Singing in the sky: Song variation in an endemic bird on the sky islands of southern India. *Anim. Behav.* **82**, 513–520. (doi:10.1016/j.anbehav.2011.05.032)
4. Oksanen, F., Blanchet, G., Kindt, R., Legendre, P., Minchin, R.B.O., Simpson, G.L., Solumos, P., Stevens, M.H.M & Wagner, H.2014 vegan: community ecology package. R package verson 2.20.http://CRAN.R-project.org/package=vegan
5. Jensen, J.L, Bohonak, A.J. & Kelley, S,T. 2005 Isolation by distance, web service. BMC Genetics 6: 13. v.3.23 <http://ibdws.sdsu.edu/>
6. Robin, V.V., Vishnudas, C.K., Gupta, P. & Ramakrishnan, U. 2015 Deep and wide valleys drive nested phylogeographic patterns across a montane bird community. *Proc. R. Soc. B* **282**: 20150861. <http://dx.doi.org/10.1098/rspb.2015.0861>
7. Robin, V.V., Gupta, P., Thatte, P. & Ramakrishnan, U. 2015 Islands within islands: two montane palaeo-endemic birds impacted by recent anthropogenic fragmentation. *Mol. Ecol.* (doi:10.1111/mec.13266)

Table S3: Summary statistics- mean ± SE (and range) - of the main spectral traits from songs of each study population.

| **Population** | **Note length** | **Phrase length** | **Notes per song** | **Song bandwidth** | **Song delivery rate** |
| --- | --- | --- | --- | --- | --- |
|  | **(seconds)** | **(seconds)** |  | **(kHz)** | **(notes/second)** |
| Brahmagiri | 0.33±0.01 (0.19-0.55) | 2.30±0.21 (0.23-9.20) | 5.10 ± 0.04  (1- 15) | 3.28±0.18  (0.86-5.60) | 2.44±0.12  (0.88-5.36) |
| Ooty | 0.36± 0.01 (0.19-0.62) | 2.5± 0.10 (1.2-5.80) | 6.7± 0.25  (3-17) | 3.6± 0.12 (0.81-6.70) | 2.8± 0.08  (0.04-50) |
| Grasshills | 0.30 ± 0.01 (0.14-0.44) | 3 ± 0.10 (0.88-8.46) | 11.14± 0.29 (3-27) | 4.90 ± 0.07 (2.05-8.40) | 3.73 ± 0.04 (2.21- 5.55) |
| Kodaikanal | 2.30±0.01 (0.1-0.39) | 3.24 ± 0.10 (0.19- 9.17) | 11.96±0.30 (2-32) | 4.85 ± 0.06 (1.44-7.15) | 3.86 ± 0.05 (0.34 -6.04) |
| Highwayvis | 0.21±0.01 (0.09-0.40) | 2.9± 0.10 (0.8-6.30) | 13.3±0.57  (3-34) | 5.5± 0.01  (2.3-7.90) | 4.7± 0.42  (2.6-7.80) |
| Peppara | 0.25± 0.01 (0.08-0.35) | 2.4± 0.13 (0.8-6.70) | 9.3± 0.36  (2-32) | 4.9±0.01  (2.6-6.90) | 4.2± 0.12  (0.94-7.60) |

Fig. S2: Spectrograms of two different WBS songs to illustrate the use of Complex Vocal Mechanisms (CVMs). Red boxes indicate pairs of notes produced within the duration of one minibreath (<0.02seconds) resulting in temporal overlap. Both songs are from different individuals from one population, in this example HWS.

Fig. S3: Significant differences between the six populations in the mean occurrence of visually identified temporal overlaps of consecutive notes per song (CVM) (Kruskal-Wallis Χ^2^ = 306.89, df = 5, p-value <0.0001, Dunn’s test results in table S3 of Supplementary Material). Error bars represent 95% confidence intervals around the mean.


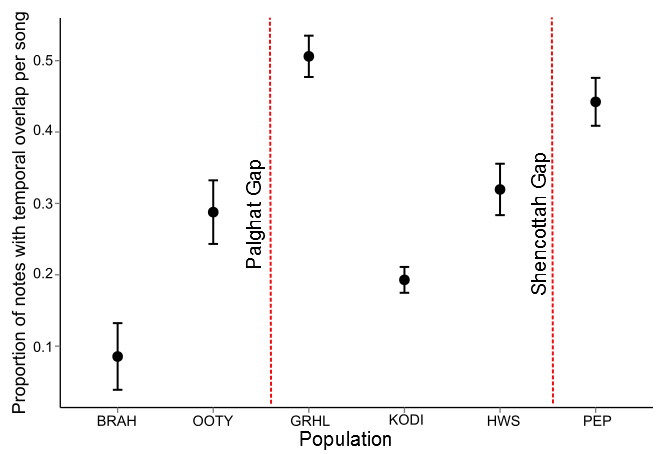


Table S4: A Dunn’s test (with a Bonferroni correction) between pairs of populations on the mean occurrence of visually identified temporal overlaps of consecutive notes per song

| **Comparison** | **Z** | **P.unadj** | **P.adj** |
| --- | --- | --- | --- |
| GRHL-BRAH=0 | 11.08 | 1.45E-28 | **2.17E-27** |
| HWS-BRAH=0 | 6.58 | 4.46E-11 | **6.68E-10** |
| HWS-GRHL=0 | -5.08 | 3.62E-07 | 5.43E-06 |
| KODI-BRAH=0 | 2.33 | 1.95E-02 | 2.92E-01 |
| KODI-GRHL=0 | -15.27 | 1.08E-52 | **1.62E-51** |
| KODI-HWS=0 | -6.75 | 1.39E-11 | **2.08E-10** |
| OOTY-BRAH=0 | 4.92 | 8.23E-07 | 1.23E-05 |
| OOTY-GRHL=0 | -7.66 | 1.83E-14 | **2.74E-13** |
| OOTY-HWS=0 | -2.13 | 3.29E-02 | 4.93E-01 |
| OOTY-KODI=0 | 4.28 | 1.79E-05 | 2.69E-04 |
| PEP-BRAH=0 | 8.76 | 1.81E-18 | **2.71E-17** |
| PEP-GRHL=0 | -2.46 | 1.39E-02 | 2.08E-01 |
| PEP-HWS=0 | 2.50 | 1.22E-02 | 1.83E-01 |
| PEP-KODI=0 | 10.33 | 4.70E-25 | **7.05E-24** |
| PEP-OOTY=0 | 4.77 | 1.78E-06 | 2.67E-05 |

Fig. S4: Non-metric multidimensional ordination of 14 spectral variables for songs of

the six colour-coded populations sampled. Songs are represented by the circles, along with 95% confidence ellipses. Stress value: 0.2056. BRAH: Brahmagiri, OOTY: Ooty, GRHL: Grasshills, KODI: Kodaikanal, HWS: Highwayvis, PEP: Peppara


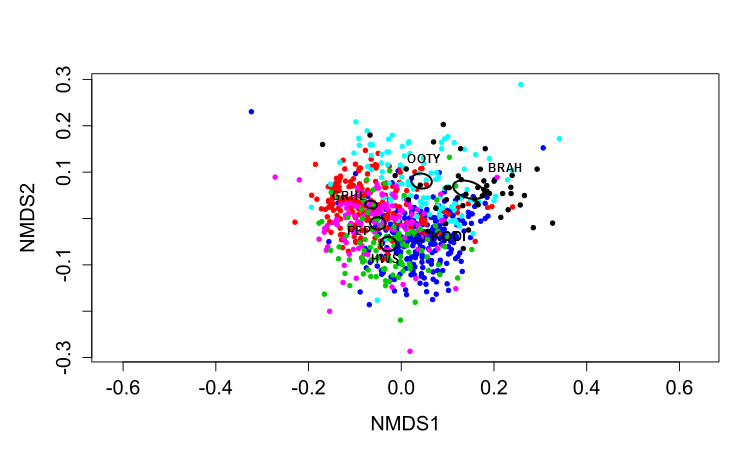


Fig. S5: Differences in versatility in song organisation between populations corresponds to the ancient Palghat gap and Shencottah gap (represented from North to South on x-axis). Error bars indicate 95% confidence intervals. Songs were categorised as new= completely new songs, ‘mod’= modified from previous songs in the same bout, ‘old’ =already sung in the bout and or ‘same’= remained unchanged from the song preceding it. BRAH: Brahmagiri, OOTY: Ooty, GRHL: Grasshills, KODI: Kodaikanal, HWS: Highwayvis, PEP: Peppara


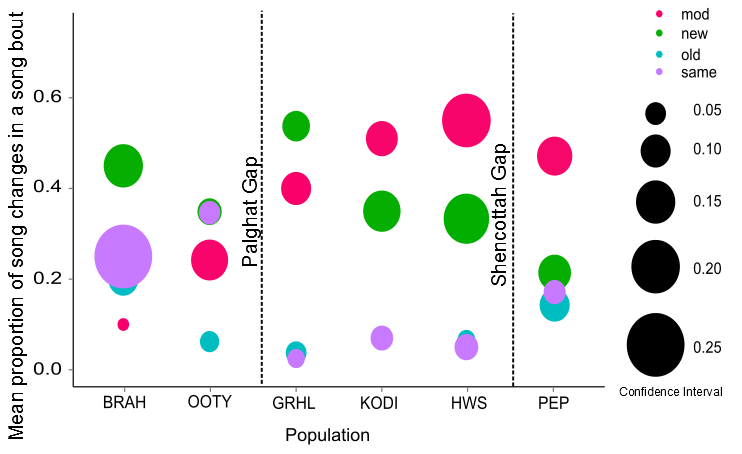

Supplement: Supplementary file 1 [file ECE3-6-7334-s001.docx]
